# Review of cigarette smoking and tuberculosis in China: intervention is needed for smoking cessation among tuberculosis patients

**DOI:** 10.1186/1471-2458-9-292

**Published:** 2009-08-12

**Authors:** Jianming Wang, Hongbing Shen

**Affiliations:** 1 Department of Epidemiology and Biostatistics, School of Public Health, Nanjing Medical University, Nanjing, PR China

## Abstract

**Background:**

As a risk factor of tuberculosis (TB), tobacco smoking has increased substantially over the past three decades, especially in developing countries. However, the association between smoking and TB, which has been shown to exist in different studies with different ethnic background, has not yet received sufficient attention in terms of TB care standards and research in China.

**Methods:**

An observational study was conducted in two rural areas of China. A total of 613 TB patients frequency matched with 1226 controls were interviewed by using a structured questionnaire. The associations between cigarette smoking and risk of TB were estimated by computing odds ratios (ORs) and 95% confidence intervals (95% CIs) from logistic regression model. Patients' smoking behavior and patterns of smoking cessation were followed after TB diagnosis. Multivariate Cox proportional hazards model was applied to calculate hazard ratios (HRs) and 95% confidence intervals (95% CIs) in analyzing the risk factors for smoking relapse. The Kaplan-Meier estimate was computed to plot the ability of smoking-free after cessation among different groups, with the Log-rank test being used to compare the difference.

**Results:**

The proportion of cigarette smoking was 54.6% in TB cases, which was significantly higher than that in controls (45.1%) with adjusted OR of 1.93(95% CI: 1.51–2.48). Though 54.9% smokers stopped smoking after being diagnosed with TB, more than 18% relapsed during the follow-up period. The proportion of relapse was higher within 6–9 months (6%) and 12–15 months (11%) after cessation. In the Cox regression estimates adjusted for age and gender, compared with those highly educated and previously treated patients, the hazard ratios of smoking relapse were 3.48(95% CI: 1.28–9.47) for less educated (< 6 years) and 4.30(95% CI: 1.01–18.30) for newly treated patients, respectively.

**Conclusion:**

Cigarette smoking is associated with TB in the Chinese. Interventions of smoking cessation are recommended to be included in the current TB control practice.

## Background

Tuberculosis (TB) is one of the leading causes of death in the world and remains a major public health burden in many developing countries [1]. From worldwide epidemic of TB in 2006, around 9.2 million new cases and 1.7 million deaths were expected every year, of which 0.7 million cases and 0.2 million deaths were in HIV-positive people [2].

As a risk factor of TB, tobacco smoking has increased substantially over the past three decades, especially in developing countries [3,4]. Globally, TB and smoking are simultaneously increasing, both of which could damage the lungs, and interact at an immunologic and cellular level [5]. Studies investigating the association between smoking and TB have been published since 1918 [6]. Both passive and active exposures to tobacco smoke have been shown to be associated with TB infection and the transition from being infected to developing TB disease. Moreover, cigarette smoking is also associated with the prognosis of TB. A cohort study conducted in Hong Kong found that significantly more current smokers developed TB and subsequently died within the follow-up period than ex-smokers and never-smokers [7]. Thomas investigated predictors of recurrence among TB patients in South India and showed that a higher relapse rate was independently associated with smoking (OR: 3.1, 95% CI: 1.6–6.0) [8].

However, the association between smoking and TB, which has been shown to exist in different studies with different ethnic background, has not yet received sufficient attention in terms of TB care standards and research. Though both smoking and TB are targeted by major international prevention and control efforts, there has been little research on the measures and effects of smoking cessation among TB patients [5].

In the present study, we aim to investigate the cigarette smoking behavior among TB patients and smoking cessation after diagnosis, and to identify factors associated with smoking relapse among quitters. It might be helpful to provide policy directions on tobacco control for current TB program.

## Methods

### Study design

**Figure 1:**
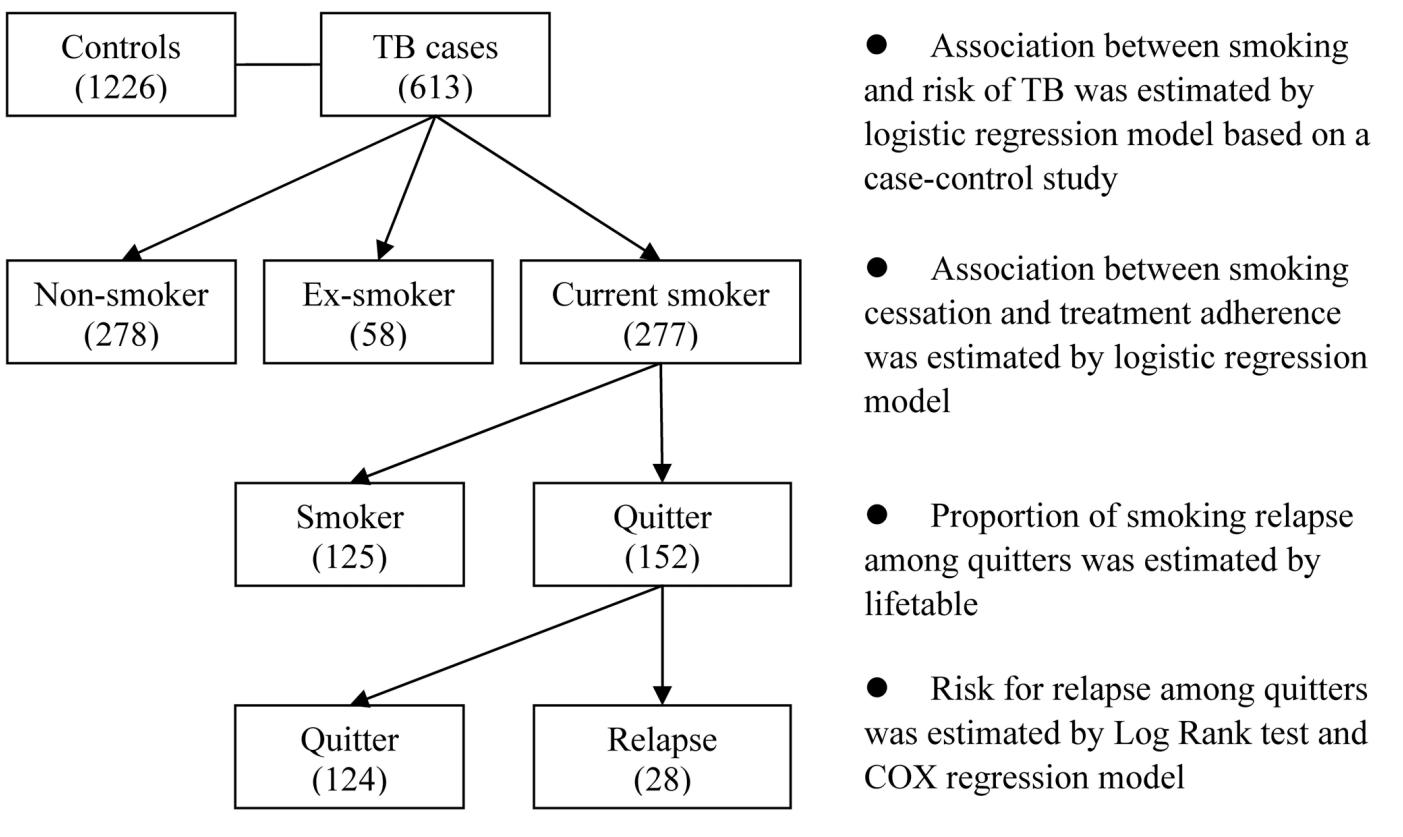
**Flow chart of the study design**.

As shown in Figure 1, a case-control study was conducted to estimate the association between cigarette smoking and risk of TB. Among current smokers, smoking cessation behavior after TB diagnosis was observed and its correlation to anti-tuberculosis treatment adherence was explored.

### Study sites

This study was conducted in Yangzhong and Wujin County, both of which were relatively rich rural areas, located in the southeast part of Jiangsu Province, China. The local TB dispensary was affiliated to the county Centre for Disease Prevention and Control (CDC) which was formerly called Anti-Epidemic Station. All newly notified TB patients were registered and managed under the DOTS (directly observed therapy, short-course) program. The annual notification rate of TB in 2007 was 79/100,000 in Yangzhong and 78/100,000 in Wujin, respectively.

### Study subjects

TB patients diagnosed in Yangzhong (905 cases from 2004 to 2007) and Wujin (1476 cases from 2006 to 2007) were target population of this study. By using simple random sampling method, 700 cases in each site were selected and those with eligible phone numbers recorded in the registry book were contacted by telephone. A total of 613 patients, including 381 patients in Yangzhong and 232 patients in Wujin County, were successfully recruited in this study. The reasons for non-response included migration, death, and stopping or changing the phone numbers, etc. To further explore the potential selection bias in this study, we compared the distribution of key characteristics between source population and these 613 cases. No significant difference was found on the distribution of gender and sputum smear test results (P > 0.05). However, the average age of patients included in this study was older than that of registered patients (56 ± 16 years vs. 50 ± 19 years). It might be attributed to the higher migration rate among young patients. A total of 1226 controls were randomly selected from a pool of more than 30,000 individuals (overall response rate: 86%) who participated in a community-based health examination program conducted in Jiangsu Province during the same time period as the cases were recruited [9]. These control subjects had no self-reported history of TB, diabetes and malignancy and were frequency matched to the cases on gender, age (± 5 years) and residential areas (urban and rural). The cases and control subjects were all genetically-unrelated Han Chinese.

### Data collection

Based on the local TB management system, all patients were followed up by TB dispensary for the whole treatment episode. To further explore the role of cigarette smoking in the risk of TB and to examine longitudinal patterns of smoking cessation among TB patients, we followed these patients again in March, 2008, by inviting them to CDC for personal interview. The interview was undertaken by trained investigators using a structured questionnaire. To ensure that all items were correctly filled in, the research supervisor double checked the questionnaires every day. For cigarette smoking status, a person who smoked at least once a day and lasted for more than 6 months in his or her lifetime was regarded as a smoker. Ex-smoker (former smoker) was defined as a smoker who had stopped smoking for at least 3 months. Smoking relapse among TB patients was defined as those who quitted smoking (≥ 3 months) after being diagnosed with TB but smoked again in the follow-up period. For alcohol drinking, it was defined as those who reported drinking alcohol more than 3 times per week. Non-adherence to anti-tuberculosis treatment was defined as those who had interrupted treatment for more than 2 weeks, including those who refused to take drugs or stopped treatment for two weeks before the end of the prescribed course.

### Data analysis

Data were managed with EpiData 3.1 (Denmark) and analyzed by STATA 10.0 (College station, TX, USA). Differences in the distribution of demographic characteristics (gender, age, education and marital status) and selected variables (alcohol drinking history) between cases and controls were tested by using the Student *t*-test (for continuous variables) or χ^2^-test (for categorical variables). The associations between cigarette smoking and risk of TB were estimated by computing odds ratios (ORs) and their 95% confidence intervals (CIs) using logistic regression model. Crude odds ratios and corresponding adjusted odds ratios by controlling age, gender and alcohol drinking history for cigarette smoking history, age of starting smoking, smoking years and cumulative pack-years were calculated respectively. To evaluate the effects of cigarette smoking on TB risk according to selected variables (gender, age, education and alcohol drinking), stratified analysis was also performed. Multivariate Cox proportional hazards model was applied to calculate hazard ratios (HRs) and 95% confidence intervals (95% CIs) in analyzing the risk factors for smoking relapse. The Kaplan-Meier estimate was computed to plot the ability of smoking-free after cessation among different groups, with the Log-rank test being used to compare the difference. During the follow-up period, if the quitter smoked again, it was defined as one event in the model; otherwise it was regarded as the censor. The criterion for significance was set at *P *< 0.05 based on a two-sided test. The continuous variables of age, age of starting smoking, smoking years, cigarettes per day and cumulative pack-year were transferred to categories by using median among controls as cutoff point.

### Ethical consideration

This project has been approved by Institutional Review Board of Nanjing Medical University. Written informed consent was obtained from all participants. Ethics has been respected throughout the whole study period.

## Results

### Cigarette smoking and risk of TB

A total of 613 patients (men 458, women 155) and 1226 healthy controls (men 916, women 310) were included in the analysis. The average age (mean ± SD) was 56.0 ± 16.5 years among cases and 56.0 ± 16.4 years among controls, respectively. As a result of frequency-matching, there were no significant differences in the distribution of gender and age between cases and controls (Table 1). The proportion of smokers was 54.6% in TB cases, which was significantly higher than that in controls (45.1%) with the crude OR of 1.47 (95% CI: 1.21–1.78). After adjusting for gender, age and alcohol drinking history, the OR was 1.93 (95% CI: 1.51–2.48). For former and current smokers, the adjusted ORs were 1.95 (95% CI: 1.32–2.87) and 1.93 (95% CI: 1.49–2.49), respectively. Both of the cumulative smoking years and amount of cigarette consumption were associated with a significantly increased risk of TB. Compared with nonsmokers, the ORs were 1.73(95% CI: 1.30–2.31) and 2.18(95% CI: 1.63–2.91) for those with cumulative pack-year less than 29 and over 29, respectively. Individuals who started smoking earlier than 25-year-old had higher risk than those who started smoking later, with the ORs of 2.09(95% CI: 1.59–2.75) and 1.72(95% CI: 1.28–2.33), respectively (Table 2). To evaluate the effects of cigarette smoking on TB risk according to selected variables, we further performed stratified analyses. As shown in Table 3, the increased risk of TB associated with smoking was more evident among the older adults (age ≥ 56 years) (OR: 2.56, 95% CI: 1.84–3.56), less educated individuals (< 6 years) (OR: 2.23, 95% CI: 1.56–3.17) and people with alcohol drinking history (OR: 2.45, 95% CI: 1.48–4.06).

**Table 1:** Basic characteristics of cases and controls

Variables	Case(n = 613) n(%)	Control(n = 1226) n(%)	*P *Value
Gender			
Men	458(74.7)	916(74.7)	1.000^†^
Women	155(25.3)	310(25.3)	
Age (years)			
Mean ± SD	56.0 ± 16.5	56.0 ± 16.4	0.963^‡^
< 56	270(44.0)	545(44.5)	0.868^†^
≥ 56	343(56.0)	681(55.5)	
Education(years)			
< 6	309(50.4)	675(55.1)	0.060^†^
≥ 6	304(49.6)	551(44.9)	
Marital status			
Single	32(5.2)	54(4.4)	0.175^†^
Married	537(87.6)	1054(86.0)	
Divorced/widowed	44(7.2)	118(9.6)	
Alcohol drinking			
Never	450(73.4)	839(68.4)	0.028^†^
Ever	163(26.6)	387(31.6)	
Sputum smear test*			
Negative	274(45.1)		
Positive	334(54.9)		

†: Chi-square test; ‡: Student *t*-test; *Five patients without sputum smear test results

**Table 2:** Association between tuberculosis and cigarette smoking

Variables	Case(n = 613) n(%)	Control(n = 1226) n(%)	cOR(95% CI)^†^	aOR(95% CI)^‡^
Cigarette smoking				
Never	278(45.4)	673(54.9)	Ref.	Ref.
Ever	335(54.6)	553(45.1)	1.47(1.21–1.78)	1.93(1.51–2.48)
Former	58(9.5)	93(7.6)	1.51(1.06–2.16)	1.95(1.32–2.87)
Current	277(45.2)	460(37.5)	1.46(1.19–1.79)	1.93(1.49–2.49)
Age of starting smoking (years)				
Never	278(45.4)	673(54.9)	Ref.	Ref.
< 25	209(34.1)	325(26.5)	1.56(1.25–1.95)	2.09(1.59–2.75)
≥ 25	126(20.5)	228(18.6)	1.34(1.03–1.73)	1.72(1.28–2.33)
Smoking years				
Never	278(45.4)	673(54.9)	Ref.	Ref.
< 30	135(22.0)	256(20.9)	1.28(0.99–1.64)	1.62(1.19–2.19)
≥ 30	200(32.6)	297(24.2)	1.63(1.30–2.05)	2.23(1.68–2.97)
Cigarettes per day				
Never	278(45.4)	673(54.9)	Ref.	Ref.
< 19	107(17.5)	204(16.6)	1.27(0.97–1.67)	1.67(1.22–2.29)
≥ 19	228(37.2)	349(28.5)	1.58(1.27–1.97)	2.08(1.59–2.72)
Cumulative pack-years				
Never	278(45.4)	673(54.9)	Ref.	Ref.
< 29	164(26.8)	299(24.4)	1.33(1.05–1.68)	1.73(1.30–2.31)
≥ 29	171(27.9)	254(20.7)	1.63(1.28–2.07)	2.18(1.63–2.91)

†cOR: crude odds ratio; ‡aOR: adjusted odds ratio, adjusting for age, gender and alcohol drinking history

**Table 3:** Stratified analysis on the association between cigarette smoking and tuberculosis by selected factors

Stratified variables	Smoking	Case(n = 613) n(%)	Control(n = 1226) n(%)	aOR(95% CI)^†^
Gender				
Men	Never	125(27.3)	366(40.0)	Ref.
	Ever	333(72.7)	550(60.0)	1.93(1.50–2.48)
Women	Never	153(98.7)	307(99.0)	Ref.
	Ever	2(1.3)	3(1.0)	2.06(0.30–14.28)
Age (years)				
< 56	Never	148(54.8)	310(56.9)	Ref.
	Ever	122(45.2)	235(43.1)	1.28(0.87–1.89)
≥ 56	Never	130(37.9)	363(53.3)	Ref.
	Ever	213(62.1)	318(46.7)	2.56(1.84–3.56)
Education (years)				
< 6	Never	142(46.0)	385(57.0)	Ref.
	Ever	167(54.0)	290(43.0)	2.23(1.56–3.17)
≥ 6	Never	136(44.7)	288(52.3)	Ref.
	Ever	168(55.3)	263(47.7)	1.68(1.18–2.38)
Alcohol drinking				
Never	Never	255(56.7)	560(66.7)	Ref.
	Ever	195(43.3)	279(33.3)	1.78(1.33–2.38)
Ever	Never	23(14.1)	113(29.2)	Ref.
	Ever	140(85.9)	274(70.8)	2.45(1.48–4.06)

†aOR: adjusted odds ratio, adjusting for age, gender and alcohol drinking history where appropriate

### Smoking cessation and treatment adherence

A total of 277 patients were current smokers at the time of diagnosis and 152 quitted smoking after being diagnosed with TB. Among those who did not stop smoking, 19.5% reported non-adherence during the treatment period, which was higher than that among quitters (11.3%) and non-smokers (15.0%). After adjusting for age, gender and alcohol drinking history, the risk for non-adherence was increased among patients without smoking cessation (OR: 2.03, 95% CI: 0.99–4.18).

### Factors associated with smoking relapse

**Figure 2:**
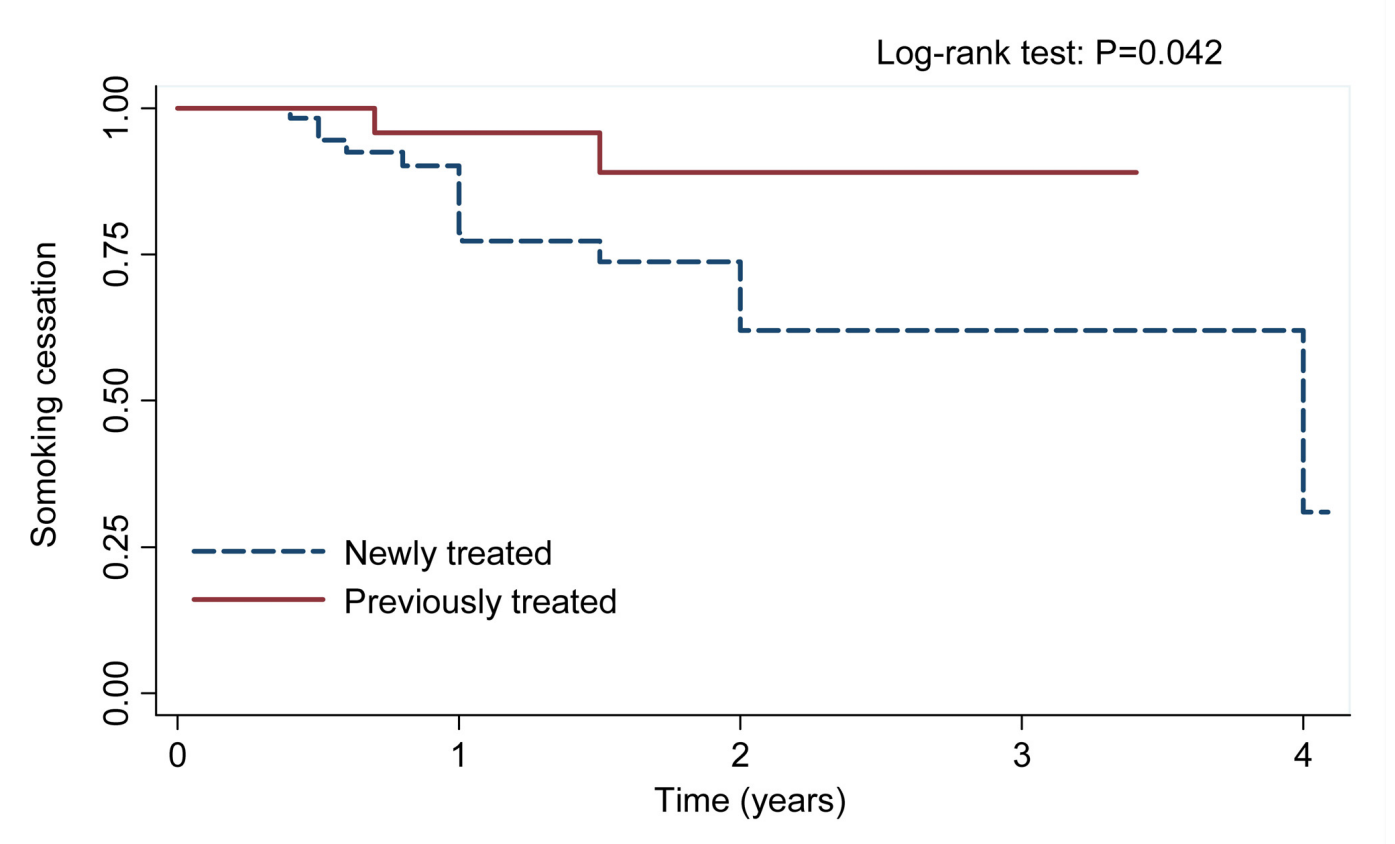
**Kaplan-Meier survival estimates for patients with different treatment history in the risk of smoking relapse**.

**Figure 3:**
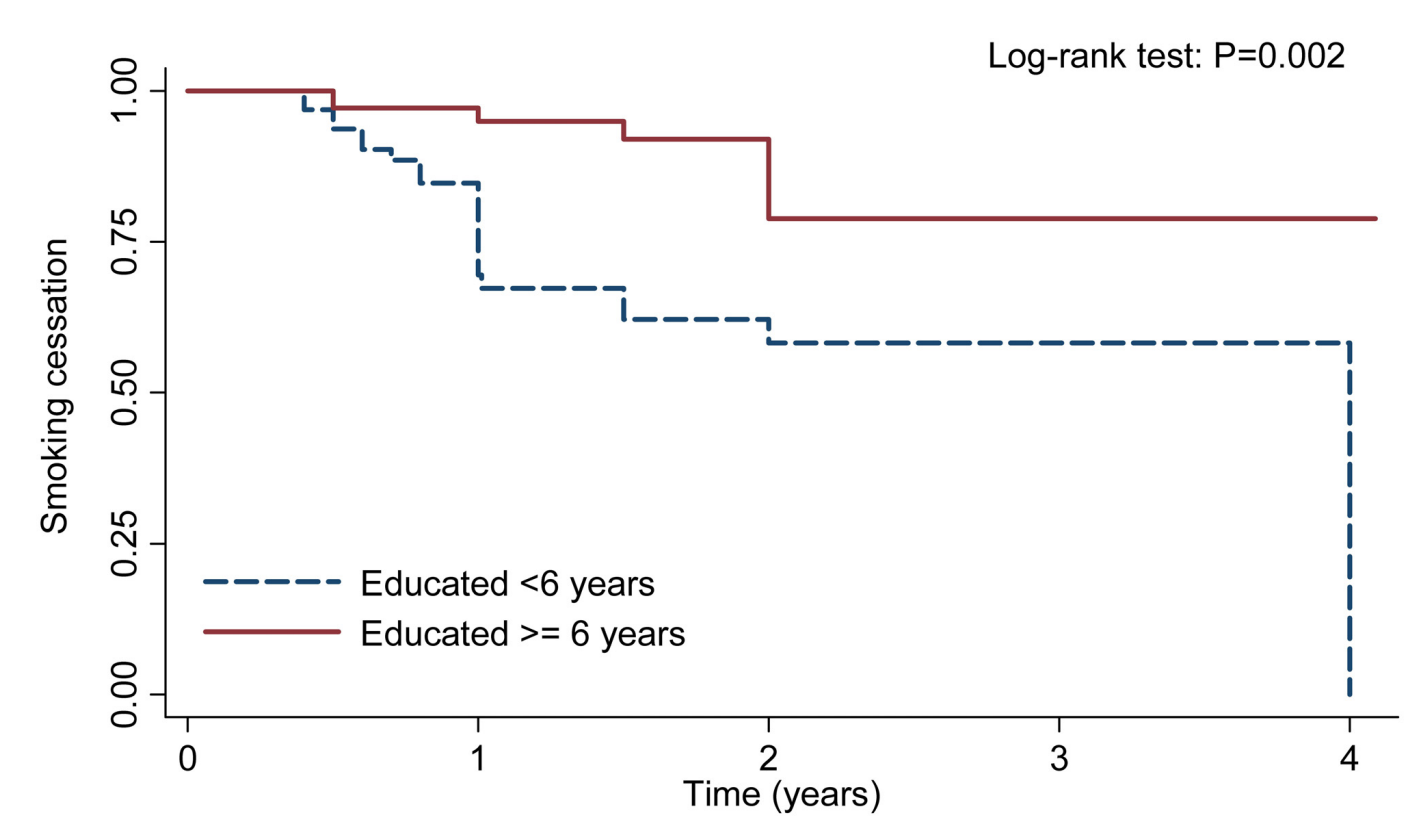
**Kaplan-Meier survival estimates for patients with different education level in the risk of smoking relapse**.

Though 54.9% smokers stopped smoking after being diagnosed with TB, more than 18% of quitters relapsed again during the follow-up period (the mean duration of follow-up was 1.35 years, ranging from 3 months to 49 months). The proportions of smoking relapse were 0, 0.01, 0.06, 0.02 and 0.11 at the time period of 0–3, 3–6, 6–9, 9–12 and 12–15 months, respectively. The Kaplan-Meier survival estimates for smoking-free after cessation among patients with different treatment history and education level were shown in Figure 2 (Log-rank test: P = 0.042) and Figure 3 (Log-rank test: P = 0.002), respectively. In the Cox regression estimates adjusted for age and gender, compared with those highly educated and previously treated patients, the hazard ratios of smoking relapse were 3.48 (95% CI: 1.28–9.47) for less educated (< 6 years) and 4.30 (95% CI: 1.01–18.30) for newly treated patients, respectively (Table 4).

**Table 4:** Factors associated with smoking relapse

Variables	Total quitters(n)	Relapse(n)	HR(95% CI)*	*P *Value
Gender				
Men	151	28	-	
Women	1	0	-	
Age (years)				
< 56	54	5	Ref.	0.779
≥ 56	98	23	1.18(0.38–3.64)	
Treatment history				
Previously treated	25	2	Ref.	0.048
Newly treated	127	26	4.30(1.01–18.30)	
Education (years)				
≥ 6	83	7	Ref.	0.014
< 6	69	21	3.48(1.28–9.47)	

*HR: adjusted hazard ratio, adjusting for age, gender, treatment history and education where appropriate

## Discussion

Apart from HIV/AIDS, tobacco smoking is the only major cause of death that is increasing rapidly [10]. It is estimated that smoking will cause about 10 million adult deaths from all causes in 2030 and most of the increased tobacco-related deaths will take place in Asia, Africa and South America [10]. China has the largest production and consumption of tobacco worldwide [11]. Although the government has announced its decision to ratify the WHO's Framework Convention on Tobacco Control and series of measures have already been taken, it is still at the beginning of its 'long way' towards improving the population's health status by reducing tobacco consumption in China [12].

Tobacco contains more than 4,500 compounds in the particulate and vapor phases which comprise five known human carcinogens and many toxic agents [13]. Long-term inhalation of tobacco smoke alters a wide range of immunological functions, resulting in significantly increased risk of heart disease, lung cancer, microbial infections and delayed recovery from these diseases [13]. Though the underlying biological mechanism is unclear, strong associations between tobacco smoking and TB have been proved in several areas [14]. Results from our study also corroborated these former reports. Furthermore, a dose-response relationship between cigarette smoking and TB was also demonstrated in the present study: with the increase of daily cigarettes consumption and duration of smoking, the risk for TB also increased accordingly.

Given that smoking is indeed causally associated with TB disease, the clinical manifestations could also be influenced by the underlying mechanism of its action [15]. A cohort of newly diagnosed TB cases were followed up from their discharge after completion of treatment and it was found that smoking was associated with the relapse of TB with OR of 2.53 (95% CI: 1.23–5.21), even after adjustment for the socioeconomic variables [16]. Although there is lack of evidence on the direct effects of smoking cessation on TB treatment outcomes, available data suggest that smokers are less adherent to TB treatment, and thus at higher risk for default and persistent infectivity [5,17]. In the present study, we also found that patients without smoking cessation had higher risk for non-adherence to anti-tuberculosis treatment. Non-adherence has been proved in several studies to contribute to prolonged infectiousness, drug resistance, relapse and death [18]. Thus, intervention for tobacco smoking is not only a public health issue among general population, but also an essential activity in TB control program.

It should be noticed that behavior of tobacco use is very difficult to change, even with medicinal aids for cessation. Only a small proportion of smokers stop smoking successfully on their own [19]. Thus smoking cessation support should be incorporated with TB control programs. As attitudes and knowledge can change, change of smoking behaviors needs to be reinforced regularly. Repeated brief cessation advice has been shown to be a feasible and inexpensive addition to routine TB case management [20]. Thus, offering advice to TB patients when they seek healthcare can influence unhealthy behaviors. The central strategy recommended by WHO for controlling TB is DOTS. However, smoking cessation has not been involved in the DOTS framework in China. TB patients lack access to smoking intervention services and social supports, resulting in lower cessation and higher relapse rate. It is essential to revise current TB treatment guidelines and provide regular medical advice on smoking behaviors involved in standard practice of DOTS and other TB control programs. However, additional research is needed in the form of pilot study to determine the feasibility of this intervention, random controlled study to test methods in various settings, and evaluation study to determine effects after widespread application of such practices [21].

One intriguing finding from the present study was that the higher risk of smoking relapse occurred in the period of 6–9 months and 12–15 months after diagnosis. It might be related with the traditional anti-tuberculosis treatment period and patient's recovery from disease. Thus interventions on smoking cessation should be focused on such special risk periods.

There are several potential limitations of this study. Firstly, our findings are based on the results of observational investigations. Though we have performed multivariate regression model and stratified analyses to control the potential confounders, other factors including economic status, intensive contact with TB patients, nutrition intake, as well as exposure to other's smoke may also confound the association between smoking and TB. Secondly, we enrolled TB patients with phone number and interviewed those who were successfully contacted through telephone. Selection bias should not be neglected. Thirdly, recall bias would also influence the results. Compared with healthy controls, TB patients could attribute disease to smoking and aggrandized it, resulting in the overestimate of smoking effects. Fourthly, a major limitation of this study was that smoking status was based on the patient's self-report rather than the detection results of nicotine levels. On one hand, some patients may decline to admit to smoking, especially if a connection between the disease and smoking has been accepted by people in the community, which will underestimate the relation between cigarette smoking and TB. On the other hand, TB patients would be more likely to falsely admit to quitting smoking for fear of disappointing the interviewers, resulting in the overestimate of smoking cessation proportion among TB patients.

## Conclusion

Cigarette smoking is associated with TB in the Chinese. Interventions of smoking cessation among patients were insufficient in rural areas of China. Physicians and DOTS providers should be actively involved in smoking cessation activities. Regular and repeated medical advices on smoking behaviors are recommended to be included in DOTS practice.

## Competing interests

The authors declare that they have no competing interests.

## Authors' contributions

JW conceived of the study, performed the statistical analysis and drafted the manuscript. HS participated in its design and coordination and helped to refine the manuscript. All authors read and approved the final manuscript.

## Pre-publication history

The pre-publication history for this paper can be accessed here:



## References

[B1] Maartens G, Wilkinson RJ (2007). Tuberculosis. Lancet.

[B2] WHO REPORT 2008 Global tuberculosis control – surveillance, planning, financing. http://www.who.int/tb/publications/global_report/2008/pdf/fullreport.pdf.

[B3] Lin HH, Ezzati M, Murray M (2007). Tobacco smoke, indoor air pollution and tuberculosis: a systematic review and meta-analysis. PLoS medicine.

[B4] Bates MN, Khalakdina A, Pai M, Chang L, Lessa F, Smith KR (2007). Risk of tuberculosis from exposure to tobacco smoke: a systematic review and meta-analysis. Archives of internal medicine.

[B5] Schneider NK, Novotny TE (2007). Addressing smoking cessation in tuberculosis control. Bulletin of the World Health Organization.

[B6] Chiang CY, Slama K, Enarson DA (2007). Associations between tobacco and tuberculosis. Int J Tuberc Lung Dis.

[B7] Leung CC, Li T, Lam TH, Yew WW, Law WS, Tam CM, Chan WM, Chan CK, Ho KS, Chang KC (2004). Smoking and tuberculosis among the elderly in Hong Kong. Am J Respir Crit Care Med.

[B8] Thomas A, Gopi PG, Santha T, Chandrasekaran V, Subramani R, Selvakumar N, Eusuff SI, Sadacharam K, Narayanan PR (2005). Predictors of relapse among pulmonary tuberculosis patients treated in a DOTS programme in South India. Int J Tuberc Lung Dis.

[B9] Chen X, Han S, Wang S, Zhou X, Zhang M, Dong J, Shi X, Qian N, Wang X, Wei Q (2009). Interactions of IL-12A and IL-12B polymorphisms on the risk of cervical cancer in Chinese women. Clin Cancer Res.

[B10] Peto R, Chen ZM, Boreham J (1999). Tobacco – the growing epidemic. Nat Med.

[B11] Zhang H, Cai B (2003). The impact of tobacco on lung health in China. Respirology.

[B12] Wang H (2006). Tobacco control in China: the dilemma between economic development and health improvement. Salud publica de Mexico.

[B13] Sopori M (2002). Effects of cigarette smoke on the immune system. Nat Rev Immunol.

[B14] Slama K, Chiang CY, Enarson DA, Hassmiller K, Fanning A, Gupta P, Ray C (2007). Tobacco and tuberculosis: a qualitative systematic review and meta-analysis. Int J Tuberc Lung Dis.

[B15] Pai M, Mohan A, Dheda K, Leung CC, Yew WW, Christopher DJ, Sharma SK (2007). Lethal interaction: the colliding epidemics of tobacco and tuberculosis. Expert review of anti-infective therapy.

[B16] d'Arc Lyra Batista J, de Fatima Pessoa Militao de Albuquerque M, de Alencar Ximenes RA, Rodrigues LC (2008). Smoking increases the risk of relapse after successful tuberculosis treatment. Int J Epidemiol.

[B17] Lavigne M, Rocher I, Steensma C, Brassard P (2006). The impact of smoking on adherence to treatment for latent tuberculosis infection. BMC Public Health.

[B18] Zignol M, Hosseini MS, Wright A, Weezenbeek CL, Nunn P, Watt CJ, Williams BG, Dye C (2006). Global incidence of multidrug-resistant tuberculosis. The Journal of infectious diseases.

[B19] Chiang CY, Slama K, Enarson DA (2007). Tobacco use and tobacco control. Int J Tuberc Lung Dis.

[B20] Slama K, Chiang CY, Enarson DA (2007). Tobacco cessation and brief advice. Int J Tuberc Lung Dis.

[B21] Novotny TE (2008). Smoking cessation and tuberculosis: connecting the DOTS. Int J Tuberc Lung Dis.

